# MiR-18a regulates myoblasts proliferation by targeting Fgf1

**DOI:** 10.1371/journal.pone.0201551

**Published:** 2018-07-31

**Authors:** Chuncheng Liu, Min Chen, Meng Wang, Wenhui Pi, Ning Li, Qingyong Meng

**Affiliations:** 1 Beijing Advanced Innovation Center for Food Nutrition and Human Health, College of Biological Science, China Agricultural University, Beijing, China; 2 The State Key Laboratory for Agrobiotechnology, College of Biological Sciences, China Agricultural University, Beijing, China; 3 College of Animal Science and Technology, China Agricultural University, Beijing, China; 4 State Key Laboratory for Sheep Genetic Improvement and Healthy Production, Xinjiang Academy of Agricultural and Reclamation Sciences, Shihezi, China; University of Massachusetts Medical School, UNITED STATES

## Abstract

MiRNAs play an important role in cell proliferation, apoptosis, and differentiation. MiR-18a is increasingly being recognized as a regulator of cancer pathogenesis. Here, we discovered that miR-18a participates in myoblasts proliferation. Expression of miR-18a was downregulated with the differentiation of C2C12 myoblasts. Overexpression of miR-18a affected the proliferation of C2C12 cells, primary myoblasts and RD cells. MiR-18a influenced the expression of cell cycle-related genes. Using TargetScan 6.2, we found that the 3’ untranslated region (UTR) of the mouse *Fgf1* gene contains complementary sequences to miR-18a. Using a siRNA, we confirmed that the reduction in the Fgf1 levels inhibited proliferation of C2C12 cells. Therefore, our results show that miR-18a participates in the regulation of proliferation by partly decreasing the expression of Fgf1.

## Introduction

MiRNAs provide a new perspective for understanding the pathway of muscle growth and differentiation. Deletion of Dicer affects normal development of mouse embryonic skeletal muscle [1, 2]. MiR-1, miR-133 [3], miR-29 [4], miR-206 [5], and miR-34 [6] regulate the proliferation and differentiation of myoblasts and satellite cells. MiR-1 [7], miR-26a [8], miR-128a [9], and miR-486 [10] regulate protein synthesis and degradation in myotubes and myofibers. These results suggest that miRNAs play an important role in skeletal muscle development.

MiR-18a belongs to the miR-17-92 cluster. Expression of this cluster is altered in various types of cancers [11, 12]. At the same time, miR-18a is also related to the occurrence and development of tumors [13]. Homozygous deletion of the miR-17-92 cluster leads to fully penetrant perinatal lethality, and these knockout mice have severe lung hypoplasia and cardiac defects [14]. MiR-18a also participates in a variety of physiological processes. MiR-18a is involved in vascular smooth muscle cells and chondrocytic differentiation by targeting syndecan4 [15] and CCN family protein 2[16], pulmonary and myocardial fibrosis *via* regulation of TGF-beta receptor II [17] and Notch 2 [18], and skeletal muscle atrophy by suppressing expression of Igf1 [19].

Fibroblast growth factors (FGFs) are known to regulate myogenic cell proliferation and differentiation [20, 21]. In the presence of FGFs and serum, MM14 skeletal muscle cells will proliferate rapidly. If the cells traverse the G1-phase in the absence of FGFs, they will permanently exit the cell cycle and embark on a terminal differentiation program [22]. FGFs can also stimulate satellite cells proliferation in mice and rats [23–25].

Here, we report that miR-18a participates in modulating proliferation in cultured C2C12 myoblasts, primary myoblasts and RD cells. MiR-18a influenced the expression of cell cycle-related genes. The 3’ UTR of the mouse *Fgf1* gene contains complementary sequences to miR-18a. Using a siRNA, we confirmed that the reduction in the Fgf1 levels inhibited proliferation of C2C12 cells. Therefore, our results show that miR-18a participates in the regulation of proliferation by partly decreasing the expression of Fgf1.

## Materials and methods

### Animals and tissue collections

Male C57BL/6 mice were purchased from the Animal Institute of the Chinese Medical Academy. These mice were raised in controlled temperatures (25 ± 1°C) and humidity (60%–70%) with a 12-hour light, 12-hour dark cycle. The mice were killed by cervical dislocation. The tibialis anterior muscle tissues were enucleated and stored at -80°C until analysis.

The skeletal muscle regeneration mouse model was induced by injecting cardiotoxin (Sigma, C9759). Cardiotoxins damages the cell membranes of skeletal muscles. Activated satellite cells in skeletal muscles can migrate, proliferate, differentiate and repair. Cardiotoxins was injected in the tibialis anterior muscle of eight-week old C57BL/6 male mice. Samples were collected at 0, 1, and 3 day(s) post cardiotoxin injection.

### Ethics statement

The experiment of animals followed the guidelines and provisions of the Use and Care of Laboratory Animals in China. The animal experiment protocol was approved by the SKLAB LABORATORY ANIMAL CARE AND USE COMMITTEE. The permission number is SKLAB-2015-01-03.

### Primary myoblast purification and culture

Primary myoblasts were isolated from hind limb muscles of 8-week-old mice. The muscles were finely minced and digested in 0.2% collagenase I (Sigma, C6885) and 2.5 U/ml dispase II (Roche, 04942078001) for 1 h, and the cell slurry was passed through a 40 mm cell filter, and then pre-plated for 4 days. Unattached cells were centrifuged and cultured on collagen-coated dishes in F10 medium (Sigma) supplemented with 20% FBS and 2.5 ng/ml fibroblast growth factor (bFGF, Invitrogen, 13256–029). After transfection, cells were cultured without bFGF.

### Cell culture

C2C12 mouse skeletal myoblasts, HEK 293T cells, and RD cells (Cell Resource Center, China) were maintained at sub-confluent densities in DMEM supplemented with 10% FBS and 1% penicillin and streptomycin. The C2C12 cell line differentiates rapidly, forming contractile myotubes and producing characteristic muscle proteins. When cells reached 80–100% confluency, myogenesis was induced by changing the medium to 2% horse serum in DMEM.

### Transfections and treatments of myoblasts

Cells were seeded in 6-well plates 24 h before transfection. The cells were transfected with 100 nM commercially synthesized miRNA mimics or with negative control (Gene Pharma, China) using Lipofectamine 2000 (Invitrogen, 11668–019) in serum-free Opti-MEM. The Opti-MEM medium was replaced with FBS-containing medium after incubation for 6 h. The sequences are as follows: miR-18a mimics forward, 5’-UAAGGUGCAUCUAGUGCAGAUAG-3’

and reverse, 5’-AUCUGCACUAGAUGCACCUUAUU-3’;

negative control forward, 5’-UUCUCCGAACGUGUCACGUTT-3’

and reverse, 5’-ACGUGACACGUUCGGAGAATT-3’;

Fgf1-c siRNA forward, 5’- CGGGCGAAGUGUAUAUAAATT-3’

and reverse, 5’- UUUAUAUACACUUCGCCCGTT-3’.

For analysis the relationship between miR-18a and Fgf1 related signal pathway, twenty-four hours after the C2C12 cells were transfected, 2.5 ng/ml bFGF or vehicle were added to the medium.

### BrdU assays

Immunohistochemistry with BrdU (Sigma, B5002) was performed using methods described previously [26, 27]. Briefly, 45 h after transfection, cells were exposed to BrdU at a final concentration of 10 μM for 3 h. Cells were then fixed with 4% paraformaldehyde and permeabilized. Samples were subsequently treated with 2 M HCl for 30 min and then with 0.01% trypsin for 12 min at 37°C. Cells were washed with PBS after each step. After blocking, the slides were incubated overnight at 4°C with anti-BrdU (Sigma, B2531). Diaminobenzidine staining was used using SPlink Detection Kits (Zhongshan, China, SP-9002) and the DAB kit (Zhongshan, China, ZLI-9018).

### Cell cycle flow cytometry

Forty-eight hours after transfection, cells were fixed in 70% ethanol overnight at 4°C, followed by incubation in 50 μg/ml propidium iodide containing 100 μg/ml RNase A and 0.2% TritonX-100 for 30 min at 4°C. The cells were analyzed in a FACSCalibur flow cytometer (BD Biosciences) and ModFit software.

### Luciferase reporter assay

HEK293T cells were cultured in DMEM supplemented with 10% FBS and 1% penicillin and streptomycin. HEK293T cells were seeded in 24-well plates. The cells were transfected using the Lipofectamine 2000 reagent with mixture A (500 ng of the psi-3’UTR-Fgf1 plasmid and 50 nM miR-18a mimics or NC) or mixture B (500 ng of psi- 3’UTR-mut-Fgf1 plasmid and 50 nM miR-18a mimics or NC). Luciferase activity was measured using the Dual Luciferase Assay System (Promega, E2920) following the manufacturer’s instructions. The data were normalized to the firefly luciferase signal.

### TaqMan^®^ miRNA expression assays

Mature miRNA expression was measured with TaqMan^®^ microRNA assays (Applied Biosystems) according to the manufacturer’s instructions. Comprehensive coverage of Sanger miRBase v10 is enabled across a two-card set of TaqMan^®^ MicroRNA Arrays (Array A and B) for a total of 518, and 303 unique assays, specific to mouse or rat miRNAs, respectively. In addition, each array contains six control assays—five carefully selected candidate endogenous control assays (three relevant to mouse, and five relevant to rat), and one negative control assay. The expression of the miRNA was normalized against the expression level of the U6 snRNA.

### Real-time quantitative PCR

Total RNA from tissue and cells was isolated using the mirVana™ miRNA Isolation Kit (Life Technologies, AM1561). The microRNA expression assay was based on a previously reported method [28]. The reverse transcriptase reactions contained the purified total RNA and 50 nM of the RT primers (miR-18a RT stem-loop primer, CTCAACTGGTGTCGTGGAGTCGGCAATTCAGTTGAGCTATCTGC; U6 RT primer CGCTTCACGAATTTGCGTGTCAT). M-MLV reverse transcriptase (Promega, 28025–013) was used. qPCR reactions were performed in triplicate. The reactions were conducted using a Roche LightCycler480 Real-Time PCR system in 96-well plates at 95°C for 10 min followed by 40 cycles of 95°C for 10 s, 57°C for 10 s and 72°C for 10 s. The primer sequences are as follows: miR-18a forward 5’-GCTGAGCTAAGGTGCATCTA G -3’ and 18a reverse 5’-TCAACTGGTGTCGTGGAGT -3’; U6 forward 5’-CTCGCTTCGGCAGCACA -3’ and

U6 reverse 5’- AACGCTTCACGAATTTGCGT -3’. U6 was used as the internal standard.

For analyzing mRNA expression, the total RNA was reverse-transcribed to cDNA using M-MLV reverse transcriptase and oligo-dT primers. qPCR reactions were performed in triplicate for each gene. The reactions were conducted using a Roche LightCycler480 Real-Time PCR system in 96-well plates at 95°C for 10 min followed by 40 cycles of 95°C for 10 s and 60°C for 1 min. The primers sequences are as follows:

Fgf1, 5’-CCAACCCAGGAGATCATTTG-3’ and 5’-ACCCAGCCTGACAGACAATC-3’;

CDK6, 5’-ATGCCGCTCTCCACCATC-3’ and 5’-GTCCGTCCGTGACACTGTG-3’;

CCND1, 5’-GCCCTCCGTATCTTACTTCAAG-3’ and 5’- ACCTCCTCTTCGCACTTCTG-3’;

GAPDH, 5’-GGCTGCCCAGAACATCAT -3’ and 5’-CGGACACATTGGGGGTAG-3’.

CDK6Homo, 5’- CCAGCAGCGGACAAATAA-3’ and 5’- ACCACAGCGTGACGACCA’;

CCND1Homo, 5’- CCGTCCATGCGGAAGATC -3’ and 5’- GGAAGCGGTCCAGGTAGTTC -3’;

GAPDH Homo, 5’-ACTCCTCCACCTTTGACGC -3’ and 5’-GCTGTAGCCAAATTCGTTGT-3’.

GAPDH was used as the internal standard.

### Western blotting

Cells were lysed using RIPA Lysis Buffer with Complete^TM^ EDTA-free protease (Roche, 05892791001). The protein concentration was determined using a BCA protein assay kit (Beyotime, China, P0012). Whole-proteome fractions from samples were subsequently separated by SDS-PAGE using 10% Tris gels, blotted onto PVDF membranes and incubated with the appropriate antibodies overnight. After incubation with relevant secondary antibodies, the labeled proteins were visualized using an ECL chemiluminescence detection kit (Merck Millipore, 34075). The following antibodies were used: anti-tubulin (Beyotime, China, AT819, 1:2000), CDK6 (Cell Signaling Technology, 3136S, 1:2000), CCND1 (Cell Signaling Technology, 2978S, 1:1000).

### ELISA assay

C2C12 cells were cultured in DMEM supplemented with 10% FBS and 1% penicillin and streptomycin. C2C12 cells were seeded in 6-well plates 24 h before transfection. The cells were transfected with 100 nM commercially synthesized miRNA mimics or with negative control (Gene Pharma, China) using Lipofectamine 2000 (Invitrogen, 11668–019) in serum-free Opti-MEM. The Opti-MEM medium was replaced with FBS-containing medium after incubation for 6 h. Twelve or eighteen hours after the cells were transfected, cells were collected and lysed using RIPA Lysis Buffer with Complete^TM^ EDTA-free protease (Roche, 05892791001). The toal protein concentration was determined using a BCA protein assay kit (Beyotime, China, P0012). The protein level of Fgf1 was determined using the ELISA kit (MSKBIO, China, KT86732).

### Vectors for psi-3’UTR-Fgf1 and psi-3’UTR-mut-Fgf1

A 914-bp fragment of the Fgf1 3’UTR, including the putative miR-18a binding sites, was amplified from mouse cDNA by PCR using KOD polymerase (TaKaRa, KOD-401). The primer sequences are as follows:

Forward 5’-CCGCTCGAGTGAAGTCTGAGACCCCAGAGA-3’ and

Reverse 5’-ATAAGAATGCGGCCGCTTAATATTGATAAGGTGCAGAGTGTAA-3’.

The amplified fragment was cloned into the Not I and Xho I sites of the psiCHECK^TM^-2 plasmid. The mutant fragment of the Fgf1 3’ UTR was amplified using the primers sequences as follows:

Fgf1-mut forward 5’- CCGCTCGAGTGAAGTCTGAGACCCCAGAGA -3’,

Fgf1-mut reverse 5’-ATAAGAATGCGGCCGCTTAATATTGAAATATTGAATACAGTAATACAG-3’;

The mutant fragment of the Fgf1 3’UTR was cloned into the psiCHECK^TM^-2 plasmid as described above.

### Statistical analysis

The results are reported as the mean ± SEM as indicated. Data were analyzed using Student’s t-test with SPSS 16.0, and p < 0.05 was considered statistically significant.

## Results

### MiR-18a participates in myogenesis

**Fig 1:**
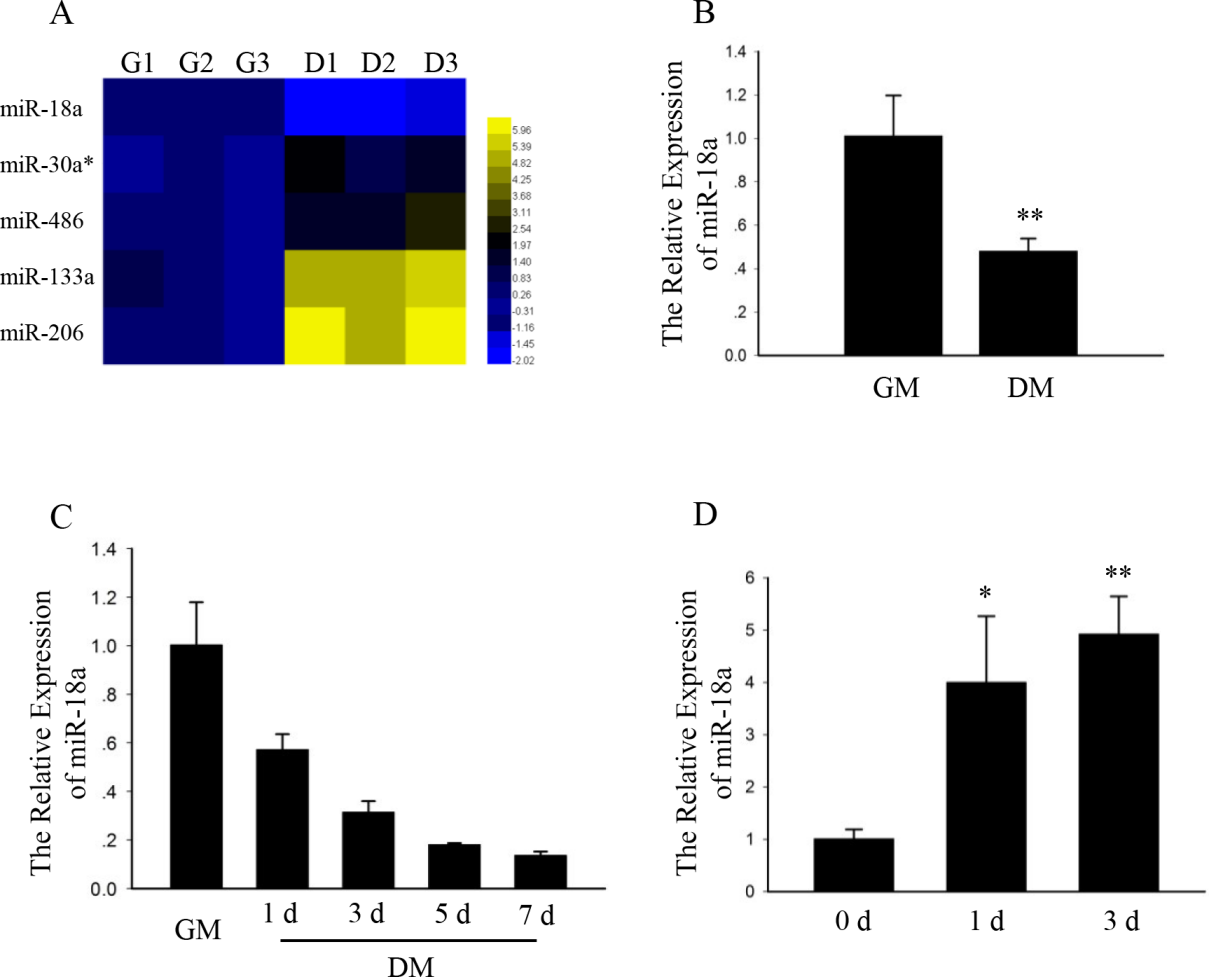
MiR-18a participates in myogenesis. (A) A heat map of significantly differentially expressed miRNAs relative to proliferating C2C12 cells. Yellow and blue represent increased and decreased expression levels, respectively. G: growth medium; D: differentiation medium for 5 days. No.1-3: various biological repetitions. (B) C2C12 myoblasts were induced to differentiate up to 5 days in differentiation medium and, at the indicated times, the relative expression of miR-18a was analyzed using qPCR with U6 small nuclear RNA as an internal reference for normalization. The values are the mean ± SEM (n = 3, biological samples). **, p < 0.01. GM: growth medium for 1 day; DM: differentiation medium for 5 days. (C) C2C12 myoblasts were induced to differentiate up to 7 days in differentiation medium and, at the indicated times, the relative expression of miR-18a was analyzed using qPCR with U6 small nuclear RNA as an internal reference for normalization. The values are the mean ± SEM (n = 3, biological samples). GM: growth medium for 1 day; DM: differentiation medium. d, day(s). (D) miR-18a levels from cardiotoxin-injected tibialis anterior muscle. MiR-18a was measured as in (B) by qPCR. n = 3 mice. *, p < 0.05; **, p < 0.01.

To understand the function of miRNAs in myoblasts proliferation and differentiation, we analyzed the expression of miRNAs during C2C12 growth and differentiation using a commercial miRNA microarray (TaqMan^®^ Rodent MicroRNA Array Set v2.0). The expression levels of miRNAs at 5 days after induction of differentiation were compared with that of proliferating myoblasts. The microarray analysis revealed 5 miRNAs were significantly changed with p values less than 0.05, normalized log_2_ greater than 1.5 or less than -1.5 (Fig 1A). Four miRNAs (miR-206, miR-133a, miR-486, and miR-30a*) were up-regulated, and one was down-regulated (miR-18a). MiR-18a were significantly differentially expressed by greater than 3-fold during the differentiation.

And we are interested in the function of miR-18a during skeletal muscle development. Expression of miR-18a in proliferating and differentiated C2C12 cells was validated by Real-time Quantitative PCR (qPCR)(Fig 1B). Then we examined the expression of miR-18a during differentiation and the levels of miR-18a steadily decreased in differentiated C2C12 cells (Fig 1C). We further detected expression of miR-18a in a cardiotoxin-injected muscle injury model and found the expression level of miR-18a to be increased during regeneration (Fig 1D). These results suggest that miR-18a is relevant in myogenesis.

To study the function of miR-18a during skeletal muscle development, we examined the expression of miR-18a in proliferating and differentiated C2C12 cells by qPCR (Fig 1B). The level of miR-18a was decreased in differentiated C2C12 cells. A time course study showed that the expression of miR-18a during differentiation and the levels of miR-18a steadily decreased in differentiated C2C12 cells (Fig 1C).

To investigate the function of miR-18a in vivo, we determined the expression of miR-18a in a cardiotoxin-injected muscle injury mouse model and found the expression level of miR-18a to be increased during regeneration (Fig 1D). These results suggest that miR-18a plays a role in myogenesis.

### MiR-18a affects C2C12 myoblasts proliferation

**Fig 2:**
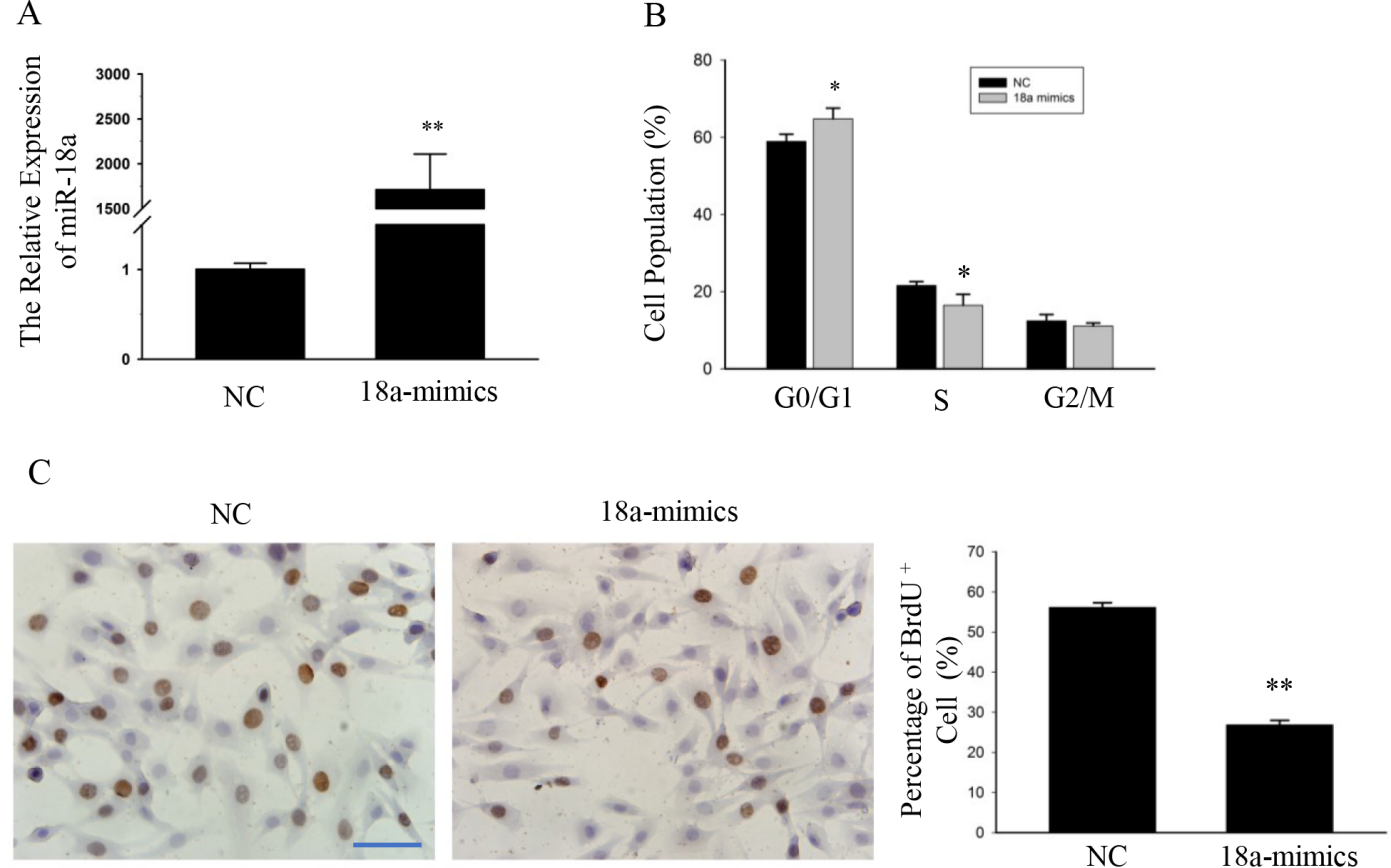
MiR-18a affected C2C12 myoblasts proliferation. (A) qPCR analysis of expression of miR-18a in C2C12 myoblasts transfected with miR-18a mimics or NC for 36 h. n = 3, biological samples. **, p < 0.01. (B) Flow cytometry was used to analyze the percentage of cells in the G0/G1, S, and G2/M phases. n = 3, biological samples. *, p < 0.05. (C) C2C12 cells were stained with BrdU. The scale bar represents 50 μm. Six images were quantitated for each group and the percentage of BrdU^+^ C2C12 cells was showed (right). **, p < 0.01.

To determine the effect of miR-18a on myoblasts proliferation, C2C12 cells were transfected with miR-18a mimics or with a negative control (NC) (Fig 2A). Forty-eight hours after transfection with miR-18a mimics, the cell cycle was analyzed by flow cytometry. The proportion of C2C12 myoblasts treated with miR-18a mimics that were in the G0/G1 phase was significantly higher than the proportion of cells transfected with NC (Fig 2B).

Furthermore, 5-bromo-2-deoxyuridine (BrdU) staining showed that miR-18a mimics reduced the number of BrdU^+^ cells by ∼50% (Fig 2C and 2D). These data confirm that miR-18a affects C2C12 myoblasts proliferation.

### MiR-18a inhibited the expression of cell cycle genes

**Fig 3:**
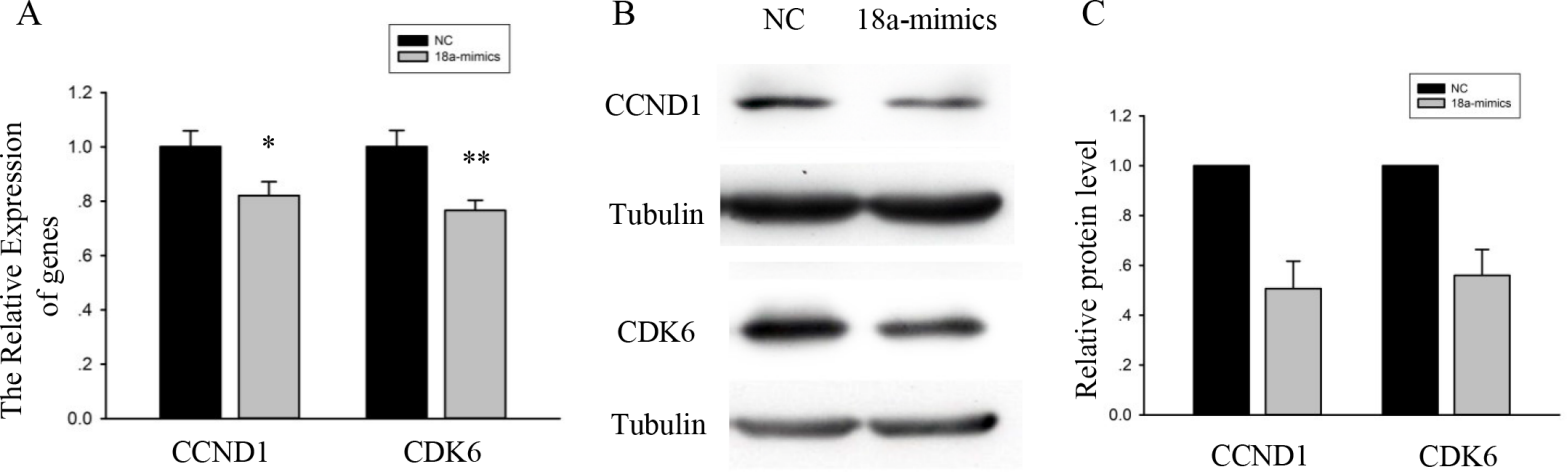
MiR-18a affected the relative expression of cell cycle genes. (A) The expression analysis for CCND1 and CDK6 in C2C12 cells transfected with miR-18a mimics or NC. GAPDH was used as an internal normalized reference. Data were shown as the means ± SEM (n = 3, biological samples). *, p < 0.05; **, p < 0.01. (B) Western blot data showed the protein level of CCND1 and CDK6 in C2C12 cells transfected with miR-18a mimics or NC. Tubulin served as the internal normalized reference. (C) Quantification of protein levels. The values are mean the ± SEM (n = 3).

To examine the function of miR-18a in the cell cycle, the expression levels of cell cycle-related genes, including Cyclin D1 (CCND1) and Cyclin Dependent Kinase 6 (CDK6), were analyzed. Compared to the myoblasts transfected with NC, qPCR analysis revealed that high levels of miR-18a were associated with low expression of CCND1 and CDK6 (Fig 3A).

As expected, according to the Western blot analysis, the protein expression levels of cell cycle genes, including CCND1 and CDK6, were reduced in C2C12 myoblasts transfected with miR-18a mimics (Fig 3B). Thus, miR-18a affects the expression of cell cycle-related genes.

### MiR-18a affected primary myoblasts and RD cells proliferation

**Fig 4:**
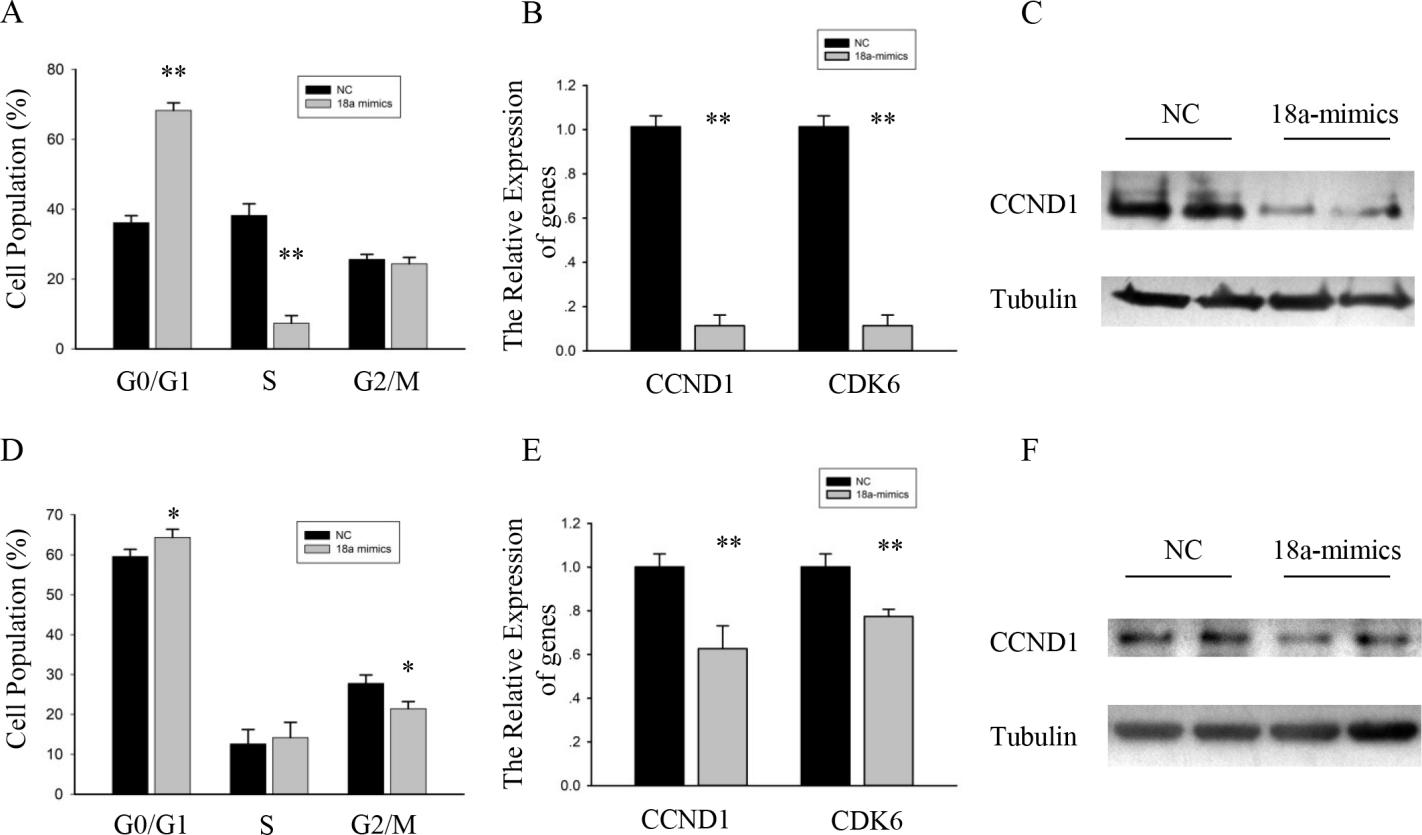
MiR-18a affected primary myoblasts and RD cells proliferation. (A) Flow cytometry was used to analyze the percentage of cells in the G0/G1, S, and G2/M phases. n = 3, biological samples. **, p < 0.01. (B) The expression analysis for CCND1 and CDK6 in primary myoblasts transfected with miR-18a mimics or NC. GAPDH was used as an internal normalized reference. Data were shown as the means ± SEM (n = 4, biological samples). **, p < 0.01. (C) Western blot data showed the protein level of CCND1 and CDK6 in primary myoblasts transfected with miR-18a mimics or NC. Tubulin served as the internal normalized reference. (D) Flow cytometry was used to analyze the percentage of cells in the G0/G1, S, and G2/M phases. n = 3, biological samples. *, p < 0.05. (E) The expression analysis for CCND1 and CDK6 in RD cells transfected with miR-18a mimics or NC. GAPDH was used as an internal normalized reference. Data were shown as the means ± SEM (n = 4, biological samples). **, p < 0.01. (F) Western blot data showed the protein level of CCND1 and CDK6 in RD cells transfected with miR-18a mimics or NC. Tubulin served as the internal normalized reference.

In addition, we assessed the function of miR-18a in primary myoblasts. We overexpressed miR-18a in primary myoblasts by transfecting miR-18a mimics. Interestingly, miR-18a has a more pronounced effect on primary myoblasts. The proportion of primary myoblasts treated with miR-18a mimics that were in the G0/G1 phase was also significantly higher than the proportion of cells transfected with NC (Fig 4A). And the mRNA and protein expression levels of cell cycle genes were reduced (Fig 4B and 4C).

miR-18a is related to the occurrence and development of tumors. To address the role of miR-18a in muscle-related rhabdomyosarcoma, we tested the effects of ectopic expression of miR-18a in the RD cell line. As similar with C2C12 myoblasts, the RD cells treated with miR-18a mimics that were in the G0/G1 phase was higher (Fig 4D). And the mRNA and protein expression levels of cell cycle genes were also reduced (Fig 4E and 4F).

Therefore, these results demonstrated that miR-18a are related with muscle related cells proliferation.

### Fgf1 was a direct target gene of miR-18a

**Fig 5:**
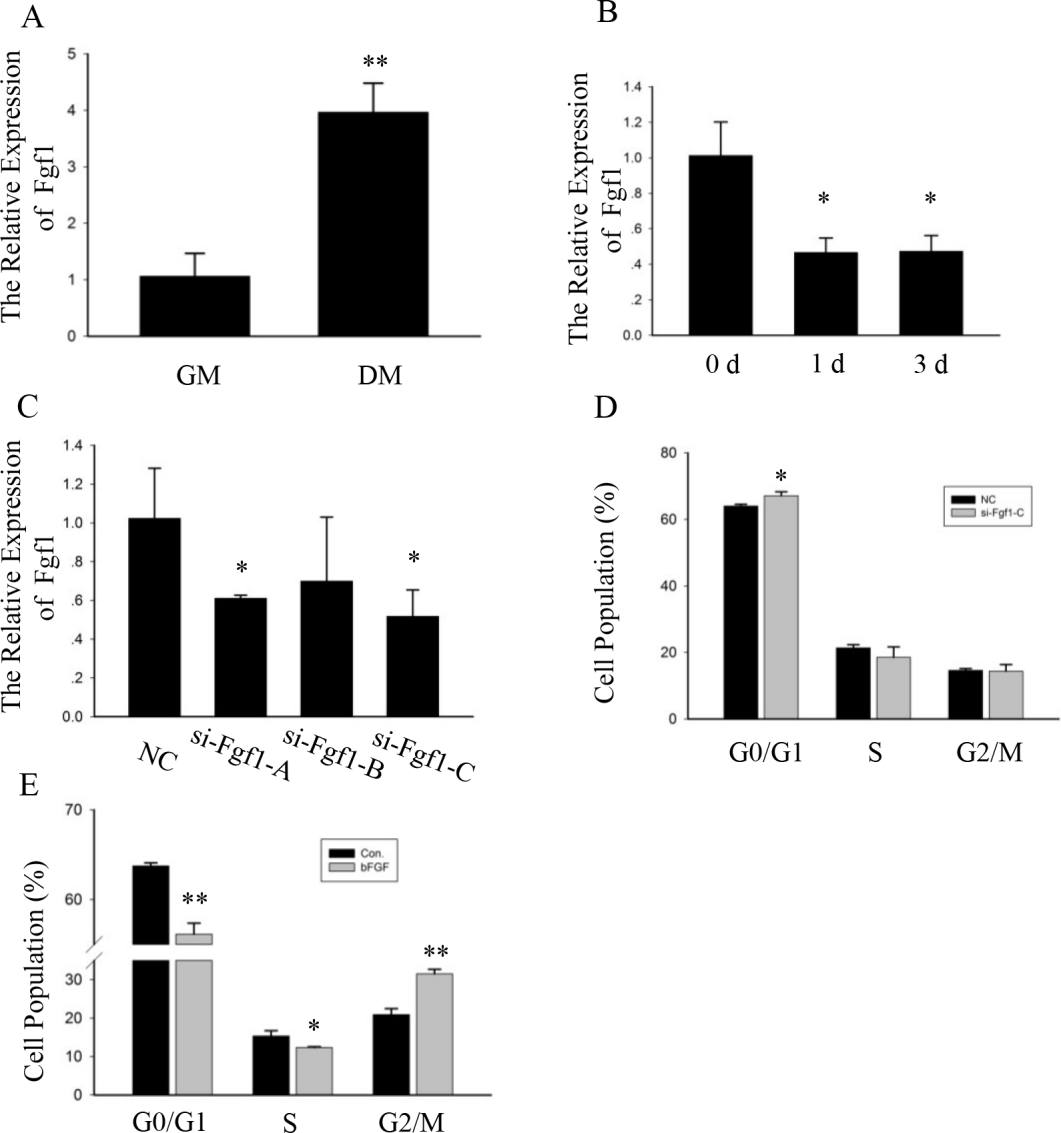
Fgf1 participated in C2C12 cells proliferation. (A) The relative expression level of Fgf1 during differentiation analyzed by qPCR and normalized to the GAPDH transcript level. The values are the mean SEM (n = 3, biological samples). **, p < 0.01. GM: growth medium for 1 day; DM: differentiation medium for 5 days. (B) Measurement of Fgf1 from cardiotoxin-injected tibialis anterior muscle. Fgf1 was measured as in (A) by qPCR. n = 3, biological samples. *, p < 0.05. d, day(s). (C) The expression analysis of Fgf1 in C2C12 transfected with si-Fgf1 A-C or NC. GAPDH served as an internal normalized reference. Data were shown as the means ± SEM (n = 3, biological samples). *, p < 0.05. (D-E) Flow cytometry was used to analyze the percentage of cells in the G0/G1, S, and G2/M phases. n = 3, biological samples. *, p < 0.05; **, p < 0.01.

We next evaluated the genes that are targeted by miR-18a. Using TargetScan 6.2, we observed that the 3’UTR of the mouse *Fgf1* gene contains complementary sequences to miR-18a. We assessed the relationship between miR-18a and Fgf1. First, we found that expression of Fgf1 was increased in differentiated C2C12 myotubes (Fig 5A), a finding that was correlated with the decreased expression of miR-18a that was previously observed after differentiation (Fig 1B). In addition, expression of Fgf1 in the skeletal muscle regeneration model was significantly decreased (Fig 5B), a finding that correlated with an increased expression of miR-18a (Fig 1D).

Fgf1 signaling is one of the best described mechanisms that regulates the cell cycle. We re-checked the role of Fgf1 in C2C12 myoblasts using either a synthetic Fgf1 siRNA or NC. The knockdown of Fgf1 was validated by qPCR (Fig 5C). In the Fgf1-knockdown cells, the proportion of cells that was increased in the G0/G1 phase compared with the proportion of cells treated with NC analyzed by flow cytometry (Fig 5D).

The basic fibroblast growth factor (bFGF) has a high binding capacity for FGF-1 receptor tyrosine kinase, so bFGF can activate Fgf1 related signal pathway. We used bFGF to test the function of Fgf1 related signal pathway on proliferation. Compared to the control, bFGF promoted C2C12 cells proliferation. Through knockdown and activation, we analyzed the effect of Fgf1 and Fgf1 related signal pathway on C2C12 myoblasts proliferation. When Fgf1 was inhibited by siRNA, cell proliferation was blocked; and when Fgf1 related signal pathway was activated by bFGF, cell proliferation was promoted. Thus, miR-18a may affect the cell cycle in C2C12 cells by suppressing expression of Fgf1 related signal pathway.

**Fig 6:**
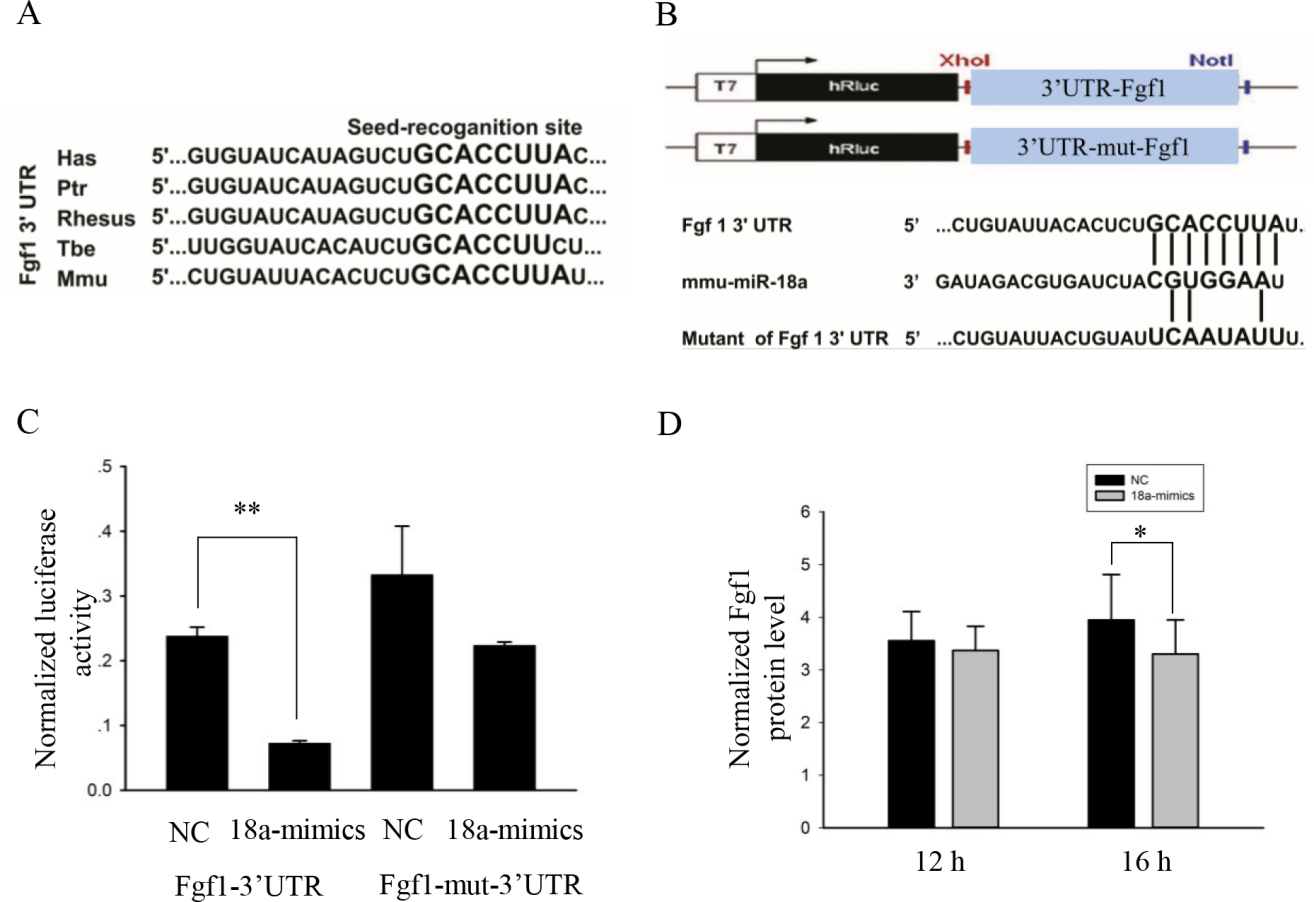
Fgf1 is a direct target gene of miR-18a. (A) The seed region of miR-18a and the seed-recognition site in the Fgf1 3′UTR are indicated in bold, and all of the nucleotides in the seed recognition sites are conserved in several species. Hsa, *Homo sapiens*; Ptr, *Pan troglodytes*; *Rhesus*, *Macaca mulatta*; Tbe, treeshrew; Mmu, *Mus musculus*. (B) A schematic of the Fgf1 3’UTR and mutant Fgf1 3’UTR sequences. (C) Relative luciferase activity detected after co-transfection of HEK293T cells with miR-18a mimics or NC and psiCHECK2^TM^ vectors. The values are the mean ± SEM. (n = 3, biological samples). **, p < 0.01. (D) ELISA showed the relative protein level of Fgf1 in C2C12 cells transfected with miR-18a mimics or NC. The total protein concentration served as the internal normalized reference. (n = 10, biological samples). *, p < 0.05.

The nucleotides in the Fgf1 3’UTR binding sites are conserved between primates and mice (Fig 6A). We used the psiCHECK^TM^-2 vector and cloned the putative 3’UTR target site downstream of the luciferase reporter gene (Fig 6B). We also constructed a reporter vector with mutations in the putative miR-18a binding sites (Fig 6B). We co-transfected the psiCHECK^TM^-2 vector (wild-type Fgf1 or mutant Fgf1) together with miR-18a mimics or NC into HEK293T cells. Forty-eight hours after transfection, the luciferase activity was measured and normalized to the activity of firefly luciferase. We found that the normalized luciferase activity of cells co-transfected with miR-18a mimics and psi-Luc-3’UTR-Fgf1 was reduced 70%. When the binding site was mutated, there was some non-specific inhibition, but only a 35% reduction. (Fig 6C). Then, we investigated whether miR-18a inhibits the expression of Fgf1 in C2C12 myoblasts. According to the ELISA (Enzyme Linked Immunosorbent Assay) analyses, miR-18a mimics decreased the expression levels of Fgf1 (Fig 6D).

**Fig 7:**
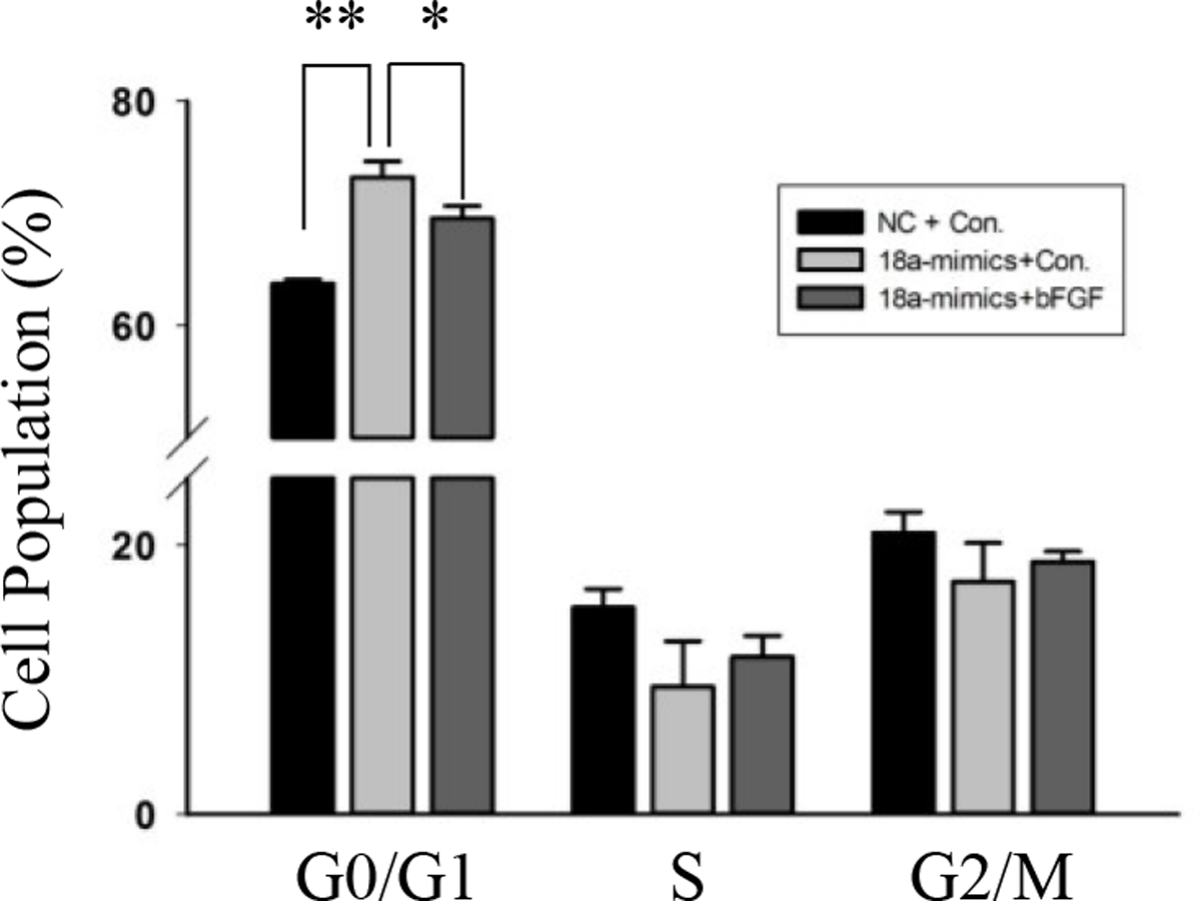
The function of miR-18 was related to Fgf1. Flow cytometry was used to analyze the percentage of cells in the G0/G1, S, and G2/M phases. n = 3, biological samples. *, p < 0.05; **, p < 0.01.

To further test the relationship between miR-18a and Fgf1 related signal pathway, C2C12 myoblasts were transfected with miR-18a mimics or NC, and bFGF or vehicle was added to the medium. The proportion of cells was assessed using flow cytometry. In the presence of bFGF, we found that the proportion of cells arrested in the G0/G1 phase was reduced (Fig 7).

## Discussion

This study established that miR-18a affected C2C12 myoblasts, primary myoblasts and RD cells proliferation. MiR-18a influenced the expression of cell cycle-related genes. The 3’ UTR of the mouse *Fgf1* gene contains complementary sequences to miR-18a. Through knockdown and activation, we analyzed the effect of Fgf1 and Fgf1 related signal pathway on C2C12 myoblasts proliferation. Therefore, our results show that miR-18a participates in the regulation of proliferation by partly decreasing the expression of Fgf1.

MiR-18a is important for skeletal muscle development. Overexpression of miR-18a induces myotubes atrophy and increases the expression of MuRF1, Atrogin-1 and Cathepsin L (CTSL). And the phosphorylation of both Akt and FoxO3 are both decreases. In this progress, miR-18a suppresses the expression of Igf1 in a 3’UTR-dependent manner[19]. In different cell types, the effect of miR-18a on proliferation varies. For example, miR-18a prevents translation of the estrogen receptor and promotes the proliferation of hepatocellular carcinoma [29]. In T24 bladder cancer cells, miR‑18a can suppress cell cycle by targeting Dicer [30]. In our study, Fgf1 was found to be a target of miR-18a in myoblasts. And as the first reported growth factor in myogenic cells, Fgf1 is mitogenic in myoblasts [21, 31].

Fgf1 is reduced in the early stages of regeneration [32, 33]. In this study, we also found expression of Fgf1 to be downregulated at the mRNA level during skeletal muscle regeneration. Fgfs are stored in the extracellular matrix [34] and are released during damage or injury [32]. Skeletal muscle regeneration requires the coordination of proliferation and differentiation. Fgf1 has been shown, both *in vitro* and *in vivo*, to stimulate proliferation and repress differentiation of myoblasts in different animals [35]. We propose that overexpression of Fgf1 may inhibit the differentiation process, and translation of Fgf1 may need to be suppressed during skeletal muscle regeneration. We found that the expression level of miR-18a increased with the regeneration. Overexpression of miR-18a can inhibit expression of Fgf1. Thus, miR-18a may also function in skeletal muscle regeneration by targeting Fgf1.

In conclusion, our study provides direct evidence that miR-18a affects C2C12 myoblasts, primary myoblasts and RD cells proliferation. We also found that the function of miR-18 was related to Fgf1. These findings suggest that miR-18a regulates the proliferation of muscle related cells.
